# Lonesome plants: How isolation affects seed set of a threatened dioecious shrub

**DOI:** 10.1002/ece3.11158

**Published:** 2024-03-21

**Authors:** Patricio García‐Guzmán, Danny E. Carvajal, Giovanni Carozzi‐Figueroa, Andrea P. Loayza

**Affiliations:** ^1^ Instituto de Ecología y Biodiversidad (IEB) Santiago Chile; ^2^ Departamento de Biología Universidad de La Serena La Serena Chile; ^3^ Facultad de Educación y Ciencias Sociales Universidad Andres Bello Santiago Chile; ^4^ Instituto Multidisciplinario de Investigación y Postgrado Universidad de La Serena La Serena Chile

**Keywords:** Atacama Desert, austral papaya, Caricaceae, conspecific neighborhood, spatial aggregation, *Vasconcellea chilensis*

## Abstract

Plant reproductive failure is a critical concern for conserving rare and endangered species that typically have low‐density and sparse populations. One important factor contributing to reproductive failure is the spatial arrangement of plants within a population, which can lead to isolation and negatively affect seed production, particularly in obligate outcrossers. Additionally, plant size can compound this effect, influencing seed production via multiple processes. Here, we investigate how spatial distribution and size influence the reproductive success of *Vasconcellea chilensis*, an endemic‐threatened papaya species in Chile. We first examined whether *V. chilensis* can produce seeds via apomixis using pollinator exclusion experiments. We then used Spatial Point Pattern Analysis (SPPA) in three populations to explore the spatial arrangement of plants. Finally, we assessed whether plant size and neighbor distance influence the reproductive success *V. chilensis* is a dioecious shrub unable to produce fruits through apomixis. The SPPA revealed significant clustering of female and male plants at different spatial scales, indicating a non‐random distribution. Moreover, a significant spatial association between the sexes was observed. In two populations, closer proximity to male plants was linked to higher seed production. Our study revealed that the reproductive system of *V. chilensis* is susceptible to distance‐dependent reproductive failure due to pollen limitation. While the species' spatial structure may partially mitigate this risk, female plants isolated from male counterparts will likely experience reduced seed set.

## Graphical abstract

Plant reproductive failure is a critical concern for conserving species that typically have low‐density and sparse populations. This study confirms that Vasconcellea chilensis is dioecious and obligate outcrosser. Significant spatial clustering was observed in female and male plants, and a significant spatial attraction between the sexes. However, these patterns were presented at different spatial scales among the populations surveyed. In two populations, proximity to male plants was directly associated with seed production but was caused by highly isolated individuals.

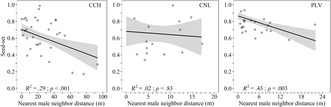

## 1 INTRODUCTION

The spatial distribution of populations, patches, and individuals is crucial to plant reproductive success (Ghazoul, 2005). Density and distance (among plants or populations) are two fundamental components of spatial distribution, and their relationship with reproductive success has traditionally been considered part of the reproductive Allee effects (Groom, 1998). For many threatened plants, lower reproductive success due to spatial isolation can be significant because such species often have narrow distributions, low population density (Mace et al., 2008; Ouborg et al., 2006), or have experienced population declines due to human‐induced habitat loss (Staude et al., 2019). Therefore, assessing the spatial distribution effects on the reproductive success of threatened plants can provide valuable insights for guiding conservation strategies within and between populations.

The underlying principle behind density‐ or distance‐dependent effects on plant reproductive success is that populations or groups of flowering individuals are less attractive to pollinators when they are found in low densities or isolated, which results in reduced levels of pollination and seed production (Alonso‐López et al., 2022; Grindeland et al., 2005). Since pollinators typically perceive flowering individuals at the within‐population scale while foraging (Lu et al., 2019; Sargent et al., 2021), pollination failure usually occurs in small populations, and Allee effects are expressed at small spatial scales (Kunin, 1997; Le Cadre et al., 2008). For instance, in a supplemental pollen experiment, Le Cadre et al. (2008) showed that pollen limitation for seed production (seeds per flower) in floral patches of *Aconitum napellus* (Ranunculaceae) increased at high isolation levels. Similarly, Brys et al. (2008) observed a negative correlation between the fruit set of *Listera ovata* (Orchidaceae) and the distance to the three nearest neighbors. Thus, pollen limitation for seed production can be highly associated with a plant's degree of spatial isolation (Ashman et al., 2004).

In sexual polymorphic species (i.e., with dioecy, gynodioecy, androdioecy, or monoecy), the relationship between pollen limitation and the degree of spatial isolation of plants is a critical issue. Due to the inability of some individuals to self‐pollinate (De Jong et al., 2005), unless they are capable of apomixis (Dupont, 2002), seed production in obligate outcrossing dioecious species often heavily relies on the spatial arrangement of mating partners. For example, the density and distance to male plants can be negatively associated with the amount of pollen carried by insects (House, 1993) and deposited on stigmas (Van Drunen & Dorken, 2012), as well as with fruit and seed production (De Jong et al., 2005; House, 1992; Van Drunen & Dorken, 2012). However, the degree to which distance‐dependent pollination limits seed production in sexual polymorphic species can vary among populations because the different sexes are not necessarily evenly distributed within populations (Castilla et al., 2012). Female plants, clustered and isolated from males, may fail to attract pollinators, ultimately compromising their seed production (Hesse & Pannell, 2011). Conversely, if male and female plants are spatially associated (sensu Ben‐Said, 2021), seed production is less likely to be limited by male plant density or distance (Erfanifard et al., 2018). Additionally, the sex ratio within populations can interact with the spatial arrangement of individuals and influence seed production. For example, a high aggregation of female plants and a low number of male plants can lead to reduced seed set (Öster & Eriksson, 2007; Timerman & Barrett, 2020; Van Drunen & Dorken, 2012).

Trait differences between sexes, alongside their spatial distribution, can significantly impact seed production. Sexual allocation theory predicts that reproductive success in female plants is resource‐limited due to their investment in flowers, fruits, and seeds, unlike male plants, which only invest in flowers (Case & Ashman, 2005; Zhang, 2006). Generally, the amount of resources a plant can store correlates with individual size (Iwasa & Kubo, 1997; Wenk & Falster, 2015), and in perennial long‐lived species, resource allocation to reproduction tends to increase with age or size (Cheplick, 2005; Samson & Werk, 1986; Wenk & Falster, 2015). Consequently, seed production in female plants is likely to be positively associated with their size. However, individual size (e.g., diameter or height) can also indirectly affect seed production via modulating other traits relevant to pollination success. For example, larger plants typically have more extensive flower displays (Albert et al., 2001; Ollerton & Lack, 1998) and may exhibit different flower phenology traits (Albert et al., 2008; Munguia‐Rosas et al., 2011; Ohya et al., 2017), both of which can enhance pollination success. Due to the potential role of individual size on seed production via multiple mechanisms, it is crucial to include it in studies examining the reproductive success of plants.

In the present study, we examined how the reproductive success of the threatened Austral papaya (*Vasconcellea chilensis*) relates to plant size and mate arrangement. This species is endemic to arid, rocky coastal ecosystems of north‐central Chile, where populations typically consist of a few isolated individuals (Ministerio de Medio Ambiente, 2008 and references therein). While there are only anecdotal accounts of the sexual system of this species, the presence of staminate and pistillate flowers suggests dioecy is likely (Arancio et al., 2001). The occurrence of asexual seed production via apomixis in this species, however, remains unexplored. Our study had three main objectives: first, to determine whether *V. chilensis* plants can produce seeds asexually through apomixis; second, to examine the spatial distribution of individuals and sexes within the populations; and third, to assess whether the size of female plants and their proximity to male plants affect reproductive success. We hypothesized that if the reproductive success of *V. chilensis* depends on mate proximity and plant size, seed set would be higher in larger plants with closer male neighbors.

## 2 MATERIALS AND METHODS

### 2.1 Study species

We studied *Vasconcellea chilensis* Planch. ex A.DC (Caricaceae), the southernmost species in the *Vasconcellea* genus. Its distribution spans from the southern edge of the Atacama region (28°39′–71°42′ W) to the Valparaiso region (33°09′ S–71°42′ W) in Chile, encompassing the southern limit of the Atacama Desert and Mediterranean coastal ecosystems. *V. chilensis* populations primarily inhabit coastal slopes, with some extending up to 30 km into inland valleys (Ministerio de Medio Ambiente, 2008). In Chile, the Austral papaya is classified as Vulnerable due to a reduction in its area of occupation (Ministerio de Medio Ambiente, 2008). This shrub can grow between 1–4 m; it has deciduous leaves and a succulent stem (Figure S1), which in some Caricaceae species has the potential to store water (Carlquist, 1998). Although various sexual systems have been proposed for *V. chilensis* (Carrasco et al., 2022), only unisexual flowers have been documented (Arancio et al., 2001). Both female (pistillate) and male (staminate) have dark red and green petals (Arancio et al., 2001) (Figure S1). Male flowers (0.7 ± 0.2 cm, *N* = 15; *personal observation*) typically grow in clusters, have nine stamens, produce nectar, and have pollen embedded in a sticky secretion. In contrast, female flowers (1.1 ± 0.3 cm, *N* = 15; *personal observation*) grow singly, are nectarless, and contain one ovary with four to nine ovules (*personal observation*). The blooming period spans late spring to early autumn (November–April) (Arancio et al., 2001; Ministerio de Medio Ambiente, 2008).

The pollination system of *V. chilensis* is unknown. Studies conducted on other members of the Caricaceae family suggest biotic pollination (Garrett, 1995), including diurnal and nocturnal insect pollinators (Bawa, 1980; Bullock & Bawa, 1981; Dey et al., 2016; Piratelli et al., 1998; Renner & Feil, 1993). We conducted pilot diurnal pollinator observations (50 hrs) in two populations (CCH and PLV; see names in the next paragraph) but did not observe any floral visitors. However, research on the sister species *V. quercifolia* indicates moth pollination (Cerino et al., 2015). *V. chilensis* bears fleshy fruits which mature from July to October, peaking in September. Each fruit contains an average of six seeds enveloped by a gelatinous layer. Dispersal agents for these seeds remain unidentified.

### 2.2 Study populations

**FIGURE 1:**
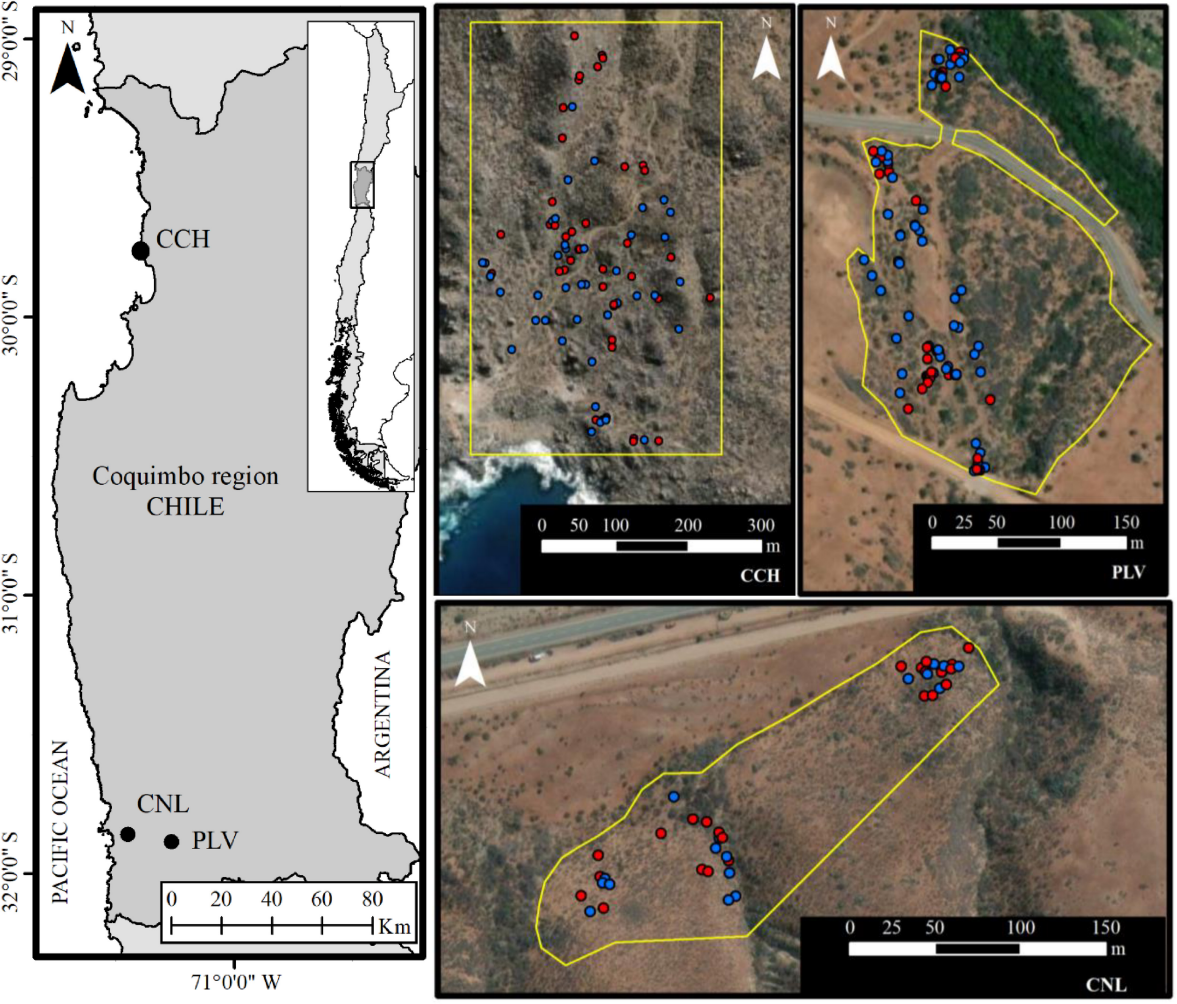
Geographic locations of the three populations studied in Chile. CCH, Conchillas; CNL, Conchalí; PLV, Puntilla Las Vacas.

We conducted our study in three populations of *V. chilensis*, named after their locations: Conchillas (CCH), Conchalí (CNL), and Puntilla Las Vacas (PLV) (Figure 1). CCH is a coastal site with little anthropogenic disturbance where *V. chilensis* grows along dried creek beds and rocky outcrops on slopes. Both CNL and PLV are situated within the Pupio Basin. In CNL, *V. chilensis* grows on north‐facing slopes bordered by old creek beds and past agricultural areas on the hill base. In PLV, *V. chilensis* grows along a dried creek bed, composed of natural habitat and areas with evidence of being impacted by past agricultural activities (Figure S1). We conducted the study during a single reproductive period (November 2021 to September 2022). The total accumulated precipitation in CCH during 2022 was 87.7 mm, with average monthly temperatures of 16.6°C during flowering and 11.6°C during fruiting. In CNL and PLV, the total accumulated precipitation during 2022 was 186 mm, and the monthly mean temperatures were 16.8°C and 11.3°C during the flowering and fruiting period, respectively (Table 1).

**TABLE 1:** Historical Mean Temperature (Tª) and Annual Accumulated Precipitation (Pp) in the study populations.

Populations	Environmental variables					
	Hist. Tª (°C) (Fl)	Obs. Tª (°C) (Fl)	Hist. Tª (°C) (Fr)	Obs. Tª (°C) (Fr)	Hist. Pp (mm)	Obs. Pp (mm)
CCH	16.7 ± 0.8	16.6 ± 1	11.8 ± 1.3	11.6 ± 0.8	81 ± 62	87.7
CNL—PLV	16.7 ± 0.2	16.8 ± 1.8	11.9 ± 1.2	11.3 ± 0.3	227 ± 159	186.5

*Note*: The table includes mean (±*SD*) Tª (1950–2022) and Pp (1965–2022) alongside observed mean Tª and Pp during flowering (Fl) (January–March 2022) and fruiting (Fr) (April–September 2022) periods. CNL and PLV data are combined due to their proximity. Source: MOP—DGA (2023).

### 2.3 Plant selection

We began by visually locating a cluster of plants in each population; then, we expanded our search within the vicinity of each group, aiming to locate additional plants. In CCH and PLV, we searched in a radius of approximately 100 m surrounding a clump or a solitary plant. In PLV and CNL, our search encompassed natural habitats and past agricultural areas. In CNL, we visually identified clusters on hills and limited our search horizontally between two creek beds, vertically from the hill base, and up to 20 m from the last plant. No additional plants were found beyond these limits.

We georeferenced all located plants using a Garmin GPS (model GPSMap 66) with the “average waypoint” function, which averages waypoints for a minimum of 5 min for increased accuracy (±3 m). For plants within 5 m of each other, distances were refined with a measuring tape. Plants with overlapping branches were recorded in the same position. From November 2021 to April 2022, we marked all male and female plants in each population (Table 2).

**TABLE 2:** Total number of *Vasconcellea chilensis* plants surveyed in each population, sex ratios, plant size, and female seed set.

	Populations		
	CCH	CNL	PLV
Total number of plants	96	41	98
Sex ratio (F/M)	1.1	1.6	0.5
Sex ratio probability	0.75	0.35	0.003
Female plant size (m^3^) (mean ± *SD*)	1.5 ± 1.6	2.8 ± 2.3	3.1 ± 3.9
Ovule number (mean ± *SD*)	7.2 ± 0.6	7.6 ± 0.8	8.5 ± 0.5
Seed set (mean ± *SD*)	0.61 ± 0.15	0.65 ± 0.17	0.8 ± 0.1

*Note*: The exact probability of having equal sex ratios (Two‐tailed binomial exact test) is also shown for each population. Population acronyms represent CCH: Conchillas; CNL: Conchalí; PLV: Puntilla Las Vacas.

### 2.4 Apomixis capacity

To determine if *V. chilensis* can produce seeds through apomixis, we covered pistillate flowers with mesh bags (mesh size 0.3 mm) to exclude pollinators and marked untreated flowers as controls. This process involved excluding one flower per plant in 15 plants in CNL and PLV and 20 in CCH (*N* = 50). We then compared the fruit production (incidences) between the control and mesh‐excluded flowers. This experiment ran from November 2021 to April 2022, with fruit production monitored until October 2022.

### 2.5 Seed production

We defined the seed set as the average number of seeds produced per total number of ovules in a flower. We calculated the mean number of ovules and seeds by counting the ovules of five flowers per plant and the seeds of at least five fruits per plant. Specifically, we counted seeds from 5–10 fruits per plant in CCH, 5–8 in CNL, and 5–17 in PLV (Figure S1). The number of flowers and fruits collected from each plant depended on their daily availability. Flower dissection for ovule counting was conducted from November 2021 to April 2022, while seed counting from collected fruits occurred between August and October 2022.

### 2.6 Plant sizes

We estimated the plant size by using canopy volume as a proxy, which is calculated as the half volume of a spheroid. This method is advantageous as it flexibly captures the irregular canopy shape of shrubs, is straightforward to measure, and offers high repeatability (Thorne et al., 2002). We first measured each female plant's height and two perpendicular diameters in the field. Then, we calculated the semi‐spheroid volume as 2/3π h/(d_1_/2 * d_2_/2), where h is the height and d_1_ and d_2_ are the diameters. These measurements were taken from November 2021 to March 2022, following the growth period 2021.

### 2.7 Spatial distribution

We evaluated the spatial distribution of plants in the three populations using a spatial point‐pattern analysis (SPPA). Firstly, we determined if the male and female plants were spatially clustered by calculating a univariate pair‐correlation function *g*(*r*) separately for each sex. Secondly, we examined if individuals of one sex were spatially associated with individuals of the opposite sex by calculating a bivariate pair‐correlation function *g*_12_(*r*). Pair correlation functions measure the probability of finding a point at a specific distance from another point in space (*r*) relative to a random distribution. The values of the pair correlation function are normalized, with a value approximating one at large distances when the points are randomly distributed (for univariate analysis) or when two‐point patterns are independent of each other (for bivariate analysis) (Wiegand & Moloney, 2014). Thus, if *g*(*r*) > 1, it indicates an aggregated distribution of plants, while if *g*(*r*) < 1, it suggests a regular distribution. Similarly, *g*_12_(*r*) > 1 indicates attraction between plants of both sexes, while if it is <1, it indicates that plants of both sexes are segregated (Ben‐Said, 2021; Wiegand & Moloney, 2004).

For our spatial analysis, we evaluated *g*(*r*) and *g*_12_(*r*) for distance classes ranging from 1 to 50 m. The resulting patterns were compared against a random pattern (i.e., null model) created from 199 simulations. In the univariate analysis, all plants were simulated as being randomly distributed across the study area (i.e., following a Complete Spatial Randomness process (CSR)). In the bivariate analysis, male and female plant patterns were also randomly distributed, but independently (i.e., following CRS Patterns 1 and 2). The random spatial simulations must be performed within defined areas (window frames). In CNL and PLV, we draw irregular polygons on satellite images as window frames because, in these populations, certain areas had been affected by past agricultural activities or intensive grazing, which lacked *V. chilensis* individuals. Hence, we excluded these areas from the window frames. On the contrary, CCH comprises natural habitats, so we did not exclude areas; instead, we utilized a rectangular window frame based on the data points. The areas of the window frames were CCH = 195,392 m^2^, CNL = 21,929 m^2^, and PLV = 46,897 m^2^. Maps illustrating the geographic distribution of plants within these window frames are shown in Figure 1.

### 2.8 Degree of isolation

To examine how the reproductive success of female plants in the three populations relates to their isolation from male plants, we calculated the degree of isolation for each female plant using two metrics: (a) the nearest male neighbor distance (NMD) and (b) the sum of the distances to the male individuals (SMD) (Ben‐Said, 2021; Wiegand et al., 2013).

### 2.9 Statistical analyses

To determine the likelihood of equal sex ratios within each population, we conducted a two‐tailed binomial exact test. To analyze the spatial distribution of plants, we used the Programita Software (Wiegand & Moloney, 2004, 2014). We assessed the differences between the observed values of *g*(*r*) and *g*_12_(*r*) with their respective random spatial patterns using global analytical envelopes. The global envelopes are analog to confidence intervals with a significance level (α) set at 0.05 (Wiegand et al., 2016). Finally, we fitted a multiple linear regression to examine how the level of spatial isolation and plant size relate to seed set. We used NMD, SMD, and plant volume as the independent variables and seed set as the dependent variable. To ensure the validity of the models, we checked for correlations among factors and their Variance Inflation Factor (VIF). If any factor had a VIF > 1/(1‐*R* ^2^), we removed the variable with the highest VIF from the model. We conducted these analyses using the *R* statistical environment (R Development Core Team, 2023).

## 3 RESULTS

### 3.1 Sexual systems and seed set

All three surveyed *V. chilensis* populations were dioecious, with plants having either staminate or pistillate flowers. The sex ratios varied among populations: CCH showed a high probability of an equal sex ratio, whereas CNL and PLV exhibited male and female‐biased ratios, respectively (Table 2). We found a positive correlation between the seed set and the number of seeds per fruit (*r* = .9; *p* < .01). Therefore, we used the seed set as a measure of seed production. The average seed set exceeded 0.6 in all sites (Table 2), with PLV having a 15% higher seed set than the other sites (One‐way ANOVA = *F*_(2,72)_ = 9.4; *p* < .01) (Figure S2).

### 3.2 Apomixis capacity

No fruits were produced by any of the bagged flowers. Conversely, on average, 88% (±13) of control flowers across all populations successfully formed fruits. These findings suggest that *V. chilensis* cannot produce fruits via apomixis.

### 3.3 Spatial distribution

**FIGURE 2:**
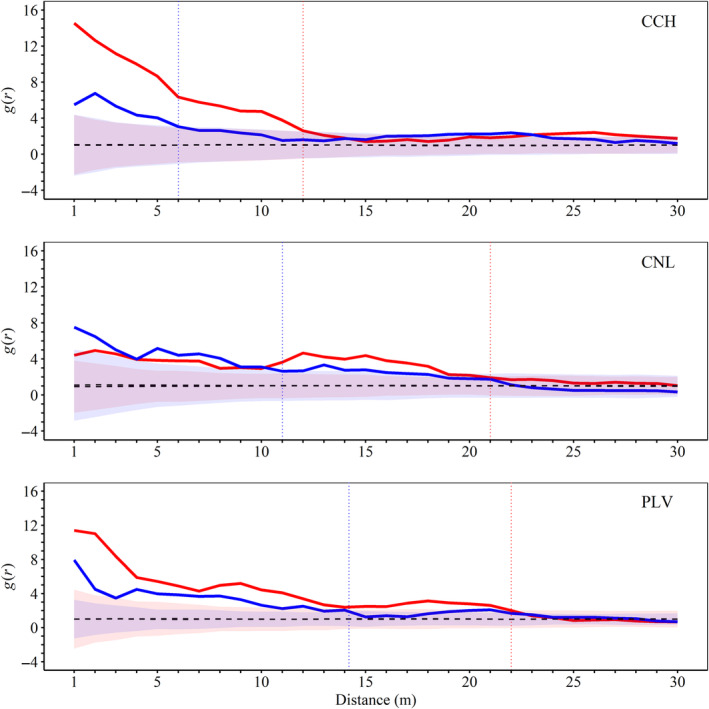
Univariate pair correlation functions *g*(*r*) for male (blue) and female (red) individuals of *Vasconcellea chilensis* in the three populations (CCH, Conchillas; CNL, Conchalí; PLV, Puntilla Las Vacas). Dashed lines represent the theoretical *g*(*r*) for a random plant distribution (null model). Global envelopes (α = 0.5) for males (light blue areas) and females (pink areas) are shown, with overlapping areas appearing purple. The dotted vertical lines indicate the distances where the observed female *g*(*r*) intersects the corresponding global envelope.

**FIGURE 3:**
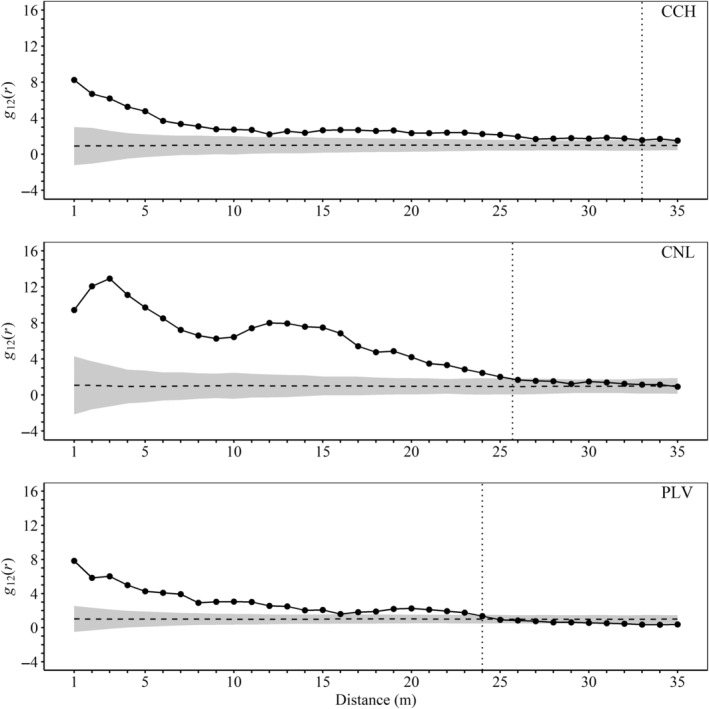
Bivariate point correlation function *g*_12_(*r*) between male and female individuals of *Vasconcellea chilensis* in the three surveyed populations (CCH, Conchillas; CNL, Conchalí; PLV, Puntilla Las Vacas). Black solid lines represent the observed *g*_12_(*r*), while dashed lines illustrate the theoretical *g*_12_(*r*) drawn for a random spatial distribution (null model). Gray areas represent the global envelope (α = 0.5) for the null models, and dotted vertical lines mark the intersection of observed *g*_12_(*r*) with the global envelope.

In all populations, *g*(*r*) values exceeded the random envelope up to varying distances for both sexes (see the vertical dotted lines in Figure 2). This indicates significant, yet differential, spatial clustering of male and female *V. chilensis* plants, with male plants showing aggregation over shorter distances compared to females. Furthermore, in all populations, *g*_12_(*r*) values also surpassed the random envelope, signifying a notable spatial association between male and female plants. CNL exhibited a greater magnitude of this association, whereas CCH showed a wider extent of association, reaching up to 33 m (Figure 3).

### 3.4 Degree of isolation

**FIGURE 4:**
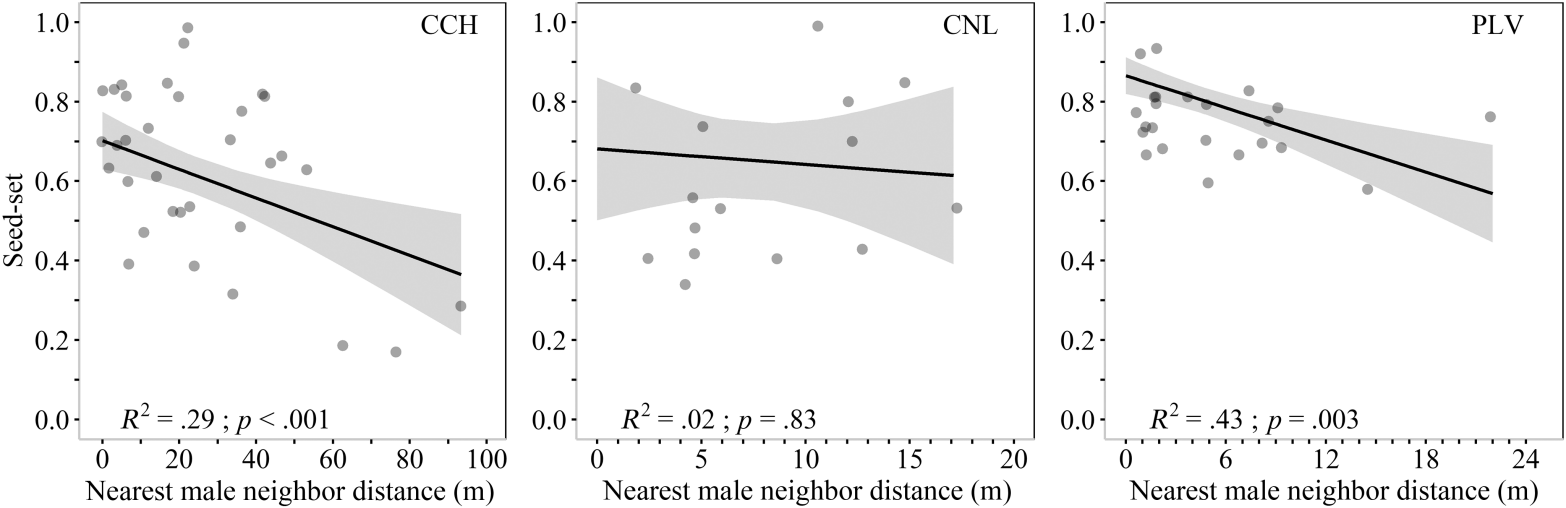
Linear relationships between seed set and the Nearest male neighbor distance in the three *Vasconcellea chilensis* populations (CCH, Conchillas; CNL, Conchalí; PLV, Puntilla Las Vacas). Predicted values of the linear models are adjusted for the mean values of covariables: (a) Plant volume = 1.5, (b) Plant volume = 2.83, (c) Plant volume = 3.13; Sum of male distances = 121. Solid black lines represent regression lines with shaded areas indicating 95% confidence intervals.

We found a strong correlation between NMD and SMD in the models fitted for CCH and CNL (Table S2). Since SMD exhibited the highest VIF in these models (Table S3), we excluded this factor and refitted the models. In the refitted models, we found a significant relationship between NMD and seed set in CCH and PLV (Figure 4; Table S4). The size of the female plants did not significantly affect their seed set in any of the fitted models (Table S4).

## 4 DISCUSSION

In the present study, we explored three key aspects of *V. chilensis*'s reproductive ecology: its potential for asexual seed set via the spatial distribution of male and female plants within populations and the influence of spatial isolation on plant reproductive success. Our findings across the three populations surveyed revealed a dioecious sexual system, with no evidence of apomictic seed production in female plants, indicating a reliance on outcrossing. We observed spatial clustering among male and female plants at various scales and significant spatial association between the sexes. Finally, in two populations, we found that the seed set in female plants decreased with increasing distance from the nearest male neighbor.

Based on the sexual morphology of flowers, we confirmed that the three surveyed populations of *V. chilensis* exhibit a dioecious sexual system. This finding contrasts with the Androdioecy and Gynodioecy systems that Carrasco et al. (2022) reported for this species. However, those authors did not provide details of their methods for sex determination or the specific populations studied. On the other hand, our observation of the absence of apomixis in *V. chilensis* aligns with previous studies on the sexual system of the Caricaceae family (Hojsgaard et al., 2014). For example, Cerino et al. (2015) reported that only 2% of fruit‐set resulted from bagged flowers in *Vasconcellea quercifolia*, while Dey et al. (2016) reported 18% of fruit set in bagged *Carica papaya* flowers. These results, along with ours, highlight the dependence of *V. chilensis* on outcross pollen, underscoring the importance of factors like spatial plant arrangement within populations for seed production. While we found no evidence of apomixis variation among or within populations in Caricaceae, its occurrence at different scales has been noted for other plant families (Bierzychudek & Eckhart, 1988; Mráz et al., 2019). Therefore, we cannot rule out the possibility of apomixis in other populations of *V. chilensis*.

Our study revealed significant spatial clustering of female plants of *V. chilensis* extending to larger spatial scales compared to males (Figure 2). Within these female clusters, we found evidence of significant spatial association with male plants (Figure 3). This pattern suggests that female plant patches are often near smaller male plant patches, potentially reducing the risk of pollen limitation (Widén & Widén, 1990), which is critical for seed set in this species, especially since female flowers of this species do not offer nectar rewards, possibly leading to reduced visitation (shorter visits seen by Cerino et al., 2015). Moreover, female flowers are generally less abundant and attract fewer visits than male or hermaphroditic flowers (Richardson et al., 2023). Although we did not directly assess pollen limitation in this study by supplementing flowers with additional pollen, the observed seed set in naturally pollinated flowers (0.6 in CCH and CNL; 0.8 in PLV; Table 2) can serve as a baseline. Using these values, we estimate a potential pollen limitation effect size of 0.5 for CCH and CNL, and 0.2 for PLV (Effect size = Ln (Seed set supplemental/Seed set control)). The effect size in PLV was at the lower end of the range reported by Dawson‐Glass and Hargreaves (2022) and lower than the 0.52 effect size reported by Knight et al. (2005). This smaller effect size in PLV, despite its weaker spatial association between sexes and smaller spatial extent compared to CCH (Figure 3), might be explained by its male‐biased sex ratio. Male‐biased populations typically face less pollen limitation due to the higher availability of male plants, increasing pollination chances for female plants (Timerman & Barrett, 2020). Thus, seed set in *V. chilensis* may be more influenced by population characteristics, such as sex ratio, than by the spatial arrangement of plants. Future studies should explore this aspect in more detail.

High spatial aggregation of *V. chilensis* plants not only impacts individual seed set but may also increase mating frequency (Robledo‐Arnuncio & Austerlitz, 2006), potentially leading to genetic structuring and reduced genetic diversity within populations (Lara‐Romero et al., 2016). However, studies by Carrasco et al. (2014, 2022) on this species report high genetic diversity within populations, a finding that seems at odds with the significant spatial aggregation we observed. A plausible explanation is that the foraging range of *V. chilensis* pollinators, which are likely moths based on findings from research on its sister species, *V. quercifolia* (Cerino et al., 2015), may extend beyond the scale of within‐population female aggregation (up to 23 m in PLV, see Figure 2). Moths from the Noctuidae (Cornet et al., 2022; Shibata & Kudo, 2023) and Sphyngidae (Lewis et al., 2023; Skogen et al., 2019) families exhibit foraging ranges from several meters to several kilometers. Thus, if *V. chilensis* is moth‐pollinated, these insects could effectively connect all plants within a population through pollen transfer, maintaining genetic diversity.

We found a relationship between NMD and female plant seed set in two of the studied populations (CCH and PLV) but not in CNL, which had lower NMD values (Figure 4). CNL's lack of association is likely due to its low NMD variation, as shown by its spatial configuration of small, mixed‐sex plant patches (Figures 1 and 4). In CCH and PLV, where the seed set was distance‐dependent, the association was predominantly driven by a few isolated plants with lower seed set. In CCH, an undisturbed site, the presence of isolated female plants could be attributed to the drier conditions, which can limit seedling recruitment to specific microhabitats (Loayza et al., 2017). This restriction and intraspecific competition for these scarce resources can lead to segregated patterns (Raventós et al., 2010), where female plants are restricted to more suitable microhabitats (Li et al., 2016). In contrast, the clustered female plants in CNL and PLV, which are disturbed sites, defy the typical pattern where disturbance increases female plant isolation, ultimately reducing seed set (Salako et al., 2016; Somanathan & Borges, 2000). Here, human activities (e.g., clearing areas for agriculture) might have contributed to increasing plant clustering by removing isolated individuals. However, to validate this hypothesis, it is necessary to compare the populations' spatial structure and seed set with an undisturbed nearby population within the Pupio Basin.

Our findings indicate that the seed set in *V. chilensis* was influenced by the Nearest Male Neighbor Distance (NMD) but not by plant size (Table S4), which we used as a proxy for stored resources. This contrasts with results from studies on species with water‐storing stems, where the stem size is often positively related to a higher seed set (Killingbeck, 2019; McIntosh, 2002). Although *V. chilensis* can store water in its stems (Carlquist, 1998), it appears that these reserves may be more critical for sustaining vegetative growth (prior flowering) and the development of fleshy fruits rather than directly contributing to seed set (Ghassemi‐Golezani et al., 2009; Killingbeck, 2019; Matthews & Shackel, 2005). Additionally, the desiccation ability of seeds in *V. chilensis* and its sister species, *V. quercifolia* (Loayza et al., 2023; Urtasun et al., 2020) suggests that water content may not be a crucial trait for early survival and growth of seedlings.

Finally, our analysis and discussion are based on the assumption of synchronized flowering between female and male *V. chilensis* plants. However, males and females often exhibit different flowering phenological traits (reviewed in Barrett & Hough, 2013, but see Cerino et al., 2015), which can lead to asynchrony within the flowering season (Munguia‐Rosas et al., 2011). This asynchrony could influence the degree of isolation and its relationship with seed set (Calabrese & Fagan, 2004). For a more comprehensive understanding of the factors affecting the reproductive success of this threatened species, future studies should explore the variation in within‐season flowering synchronization between sexes and its potential impact on female plant isolation. Additionally, the relation between asynchrony and spatial isolation should be examined in the context of ENSO effects on inter‐annual precipitation variability, given that flowering and flowering synchrony in arid systems can depend on precipitation (Vidiella et al., 1999).

## 5 CONCLUSIONS

Our study has shown that *V. chilensis* is highly vulnerable to distance‐dependent reproductive failure, stemming from its inability to reproduce asexually via apomixis. The spatial proximity of female and male plants could alleviate this risk for most individuals, but isolated plants without nearby male neighbors are likely to have lower seed set. Despite most plants being near potential mates, maintaining this spatial structure is crucial, especially considering the ongoing habitat loss affecting some populations of this species.

## AUTHOR CONTRIBUTIONS

**Patricio García‐Guzmán:** Conceptualization (equal); data curation (equal); formal analysis (equal); investigation (equal); methodology (equal); software (equal); visualization (equal); writing – original draft (equal); writing – review and editing (equal). **Danny E. Carvajal:** Investigation (equal). **Giovanni Carozzi‐Figueroa:** Investigation (equal). **Andrea P. Loayza:** Funding acquisition (equal); project administration (equal); resources (equal); supervision (equal); writing – review and editing (equal).

## Supporting information


Appendix S1.

Appendix S2.

## Data Availability

Data for SPPA's analyses, Programita Software parameters, and R code used for graphs and analyses are available at Dryad (https://datadryad.org/stash/share/OUX2sBwU7g8ee8FIcyFsU1EGnhmZJInlvGNACZAYXBw).
