# Supplementary material for: Lonesome plants: How isolation affects seed set of a threatened dioecious shrub
Source: Ecol Evol. 2024 Mar 21;14(3):e11158. doi: 10.1002/ece3.11158 (PMC10955460; doi:10.1002/ece3.11158)
Supplement: Supplementary file 1 — Appendix S1. [file ECE3-14-e11158-s001.pdf]

APPENDIX 1

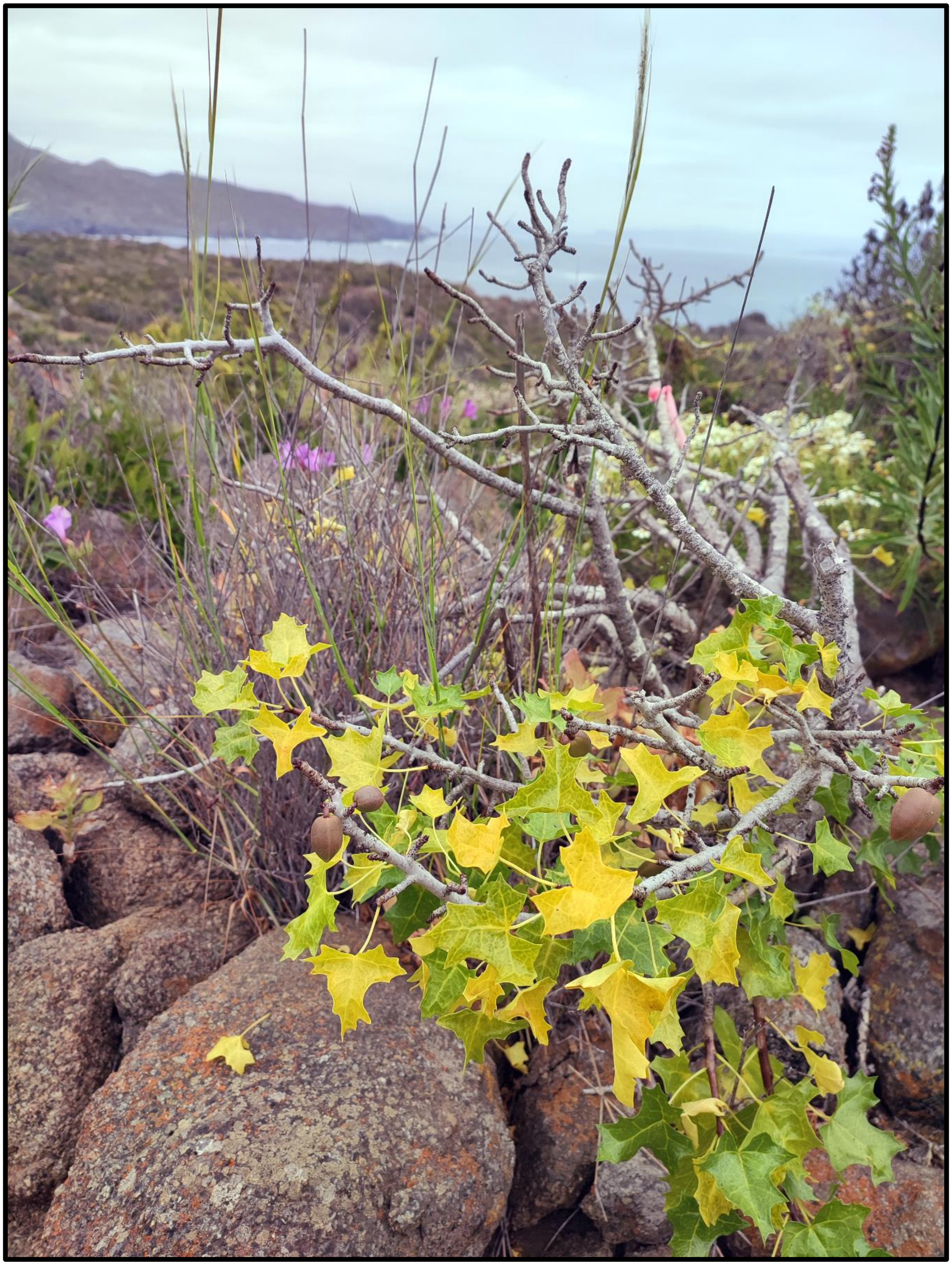

Figure S1: Female plant of *Vasconcellea chilensis* growing in its natural habitat. Conchillas (29°46’14” S – 71°19’49” O), Chile.

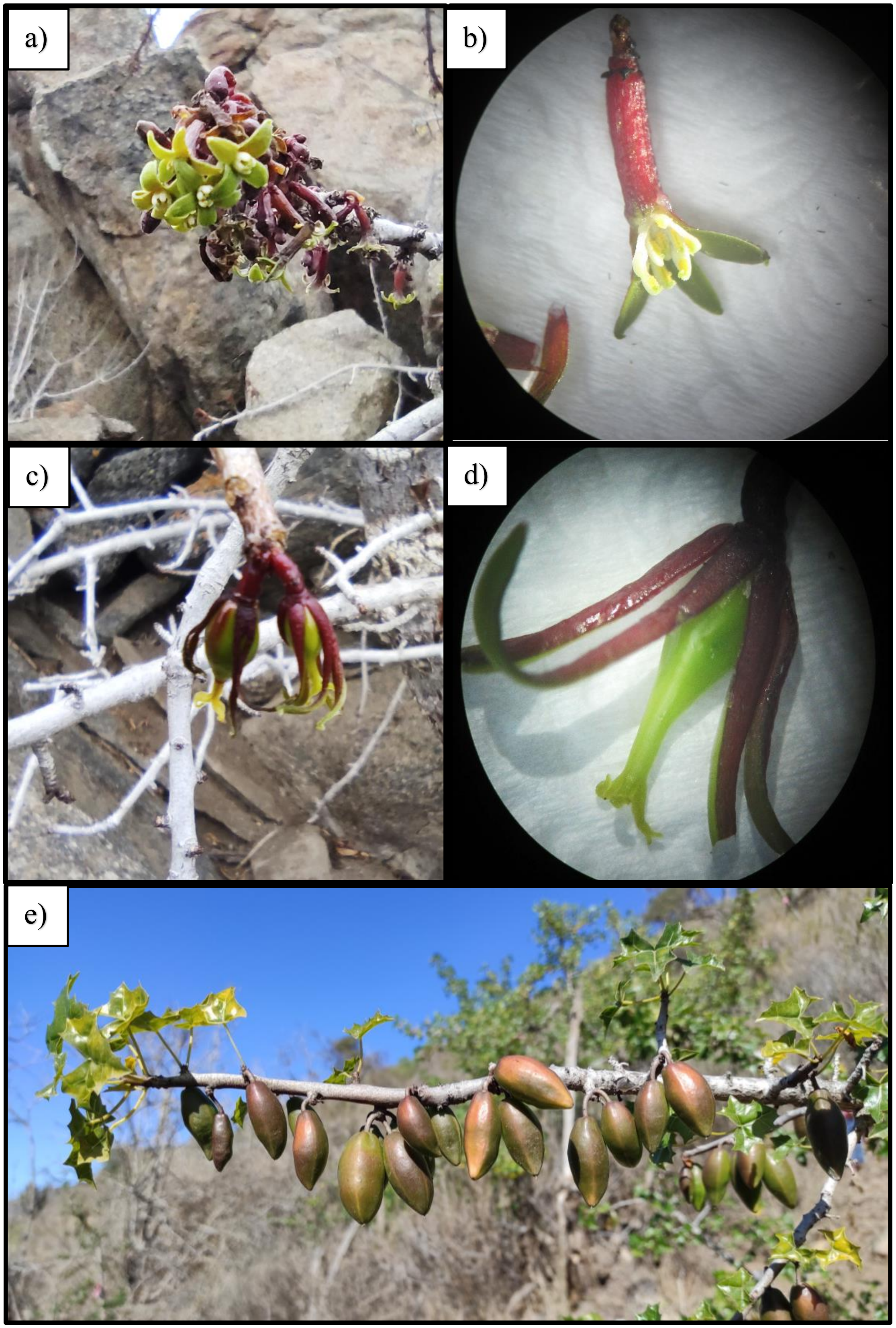

Figure S2: Reproductive organs of *Vasconcellea chilensis.* a) Male (staminate) flower, b) dissected male flower showing stamens, c) female (pistillate) flower, d) female dissected flower showing pistil and ovary and e) fruits.

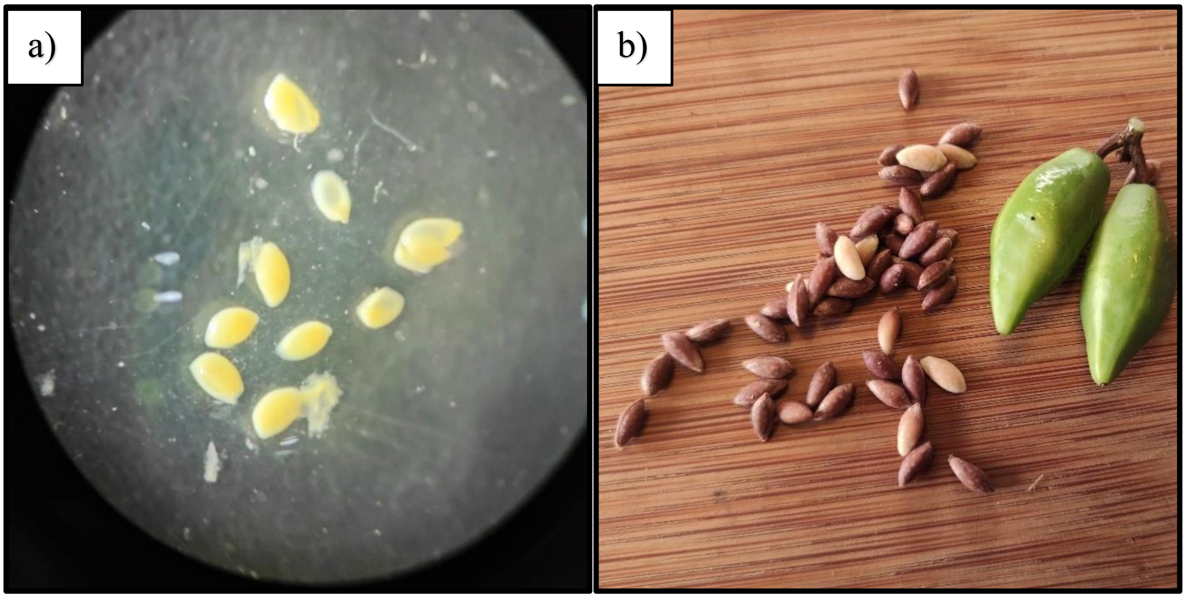

Figure S3: Reproductive organs of *Vasconcellea chilensis.* a) Ovules dissected from the ovary and b) seeds separated from the fruits.

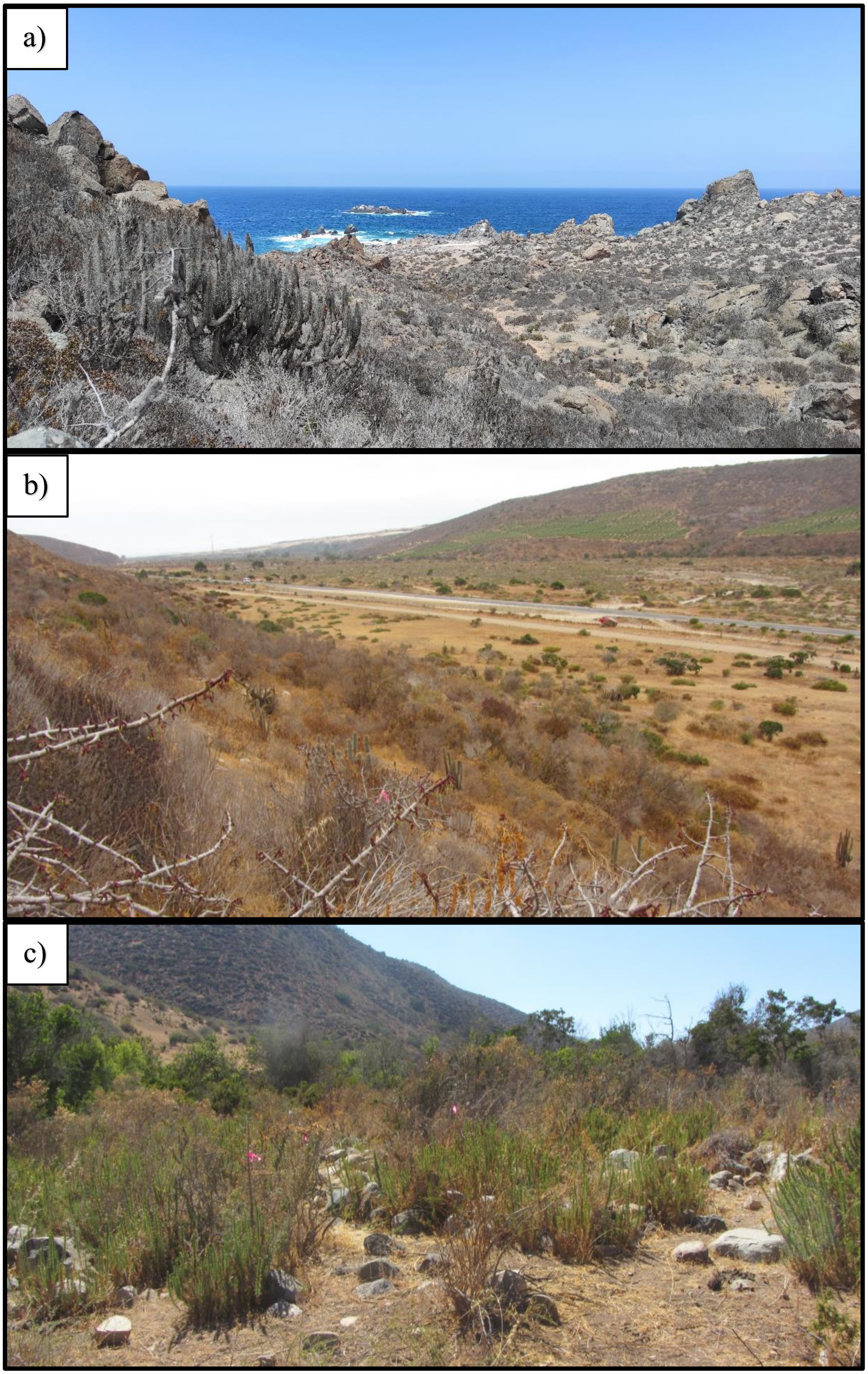

Figure S4: General view of the studied populations a) Conchillas (CCH), b) Conchalí (CNL) and c) Puntilla Las Vacas (PLV). Photos were taken during the flowering period (December to March).
