# Supplementary material for: Lonesome plants: How isolation affects seed set of a threatened dioecious shrub
Source: Ecol Evol. 2024 Mar 21;14(3):e11158. doi: 10.1002/ece3.11158 (PMC10955460; doi:10.1002/ece3.11158)
Supplement: Supplementary file 2 — Appendix S2. [file ECE3-14-e11158-s002.docx]

**APPENDIX 2**

**
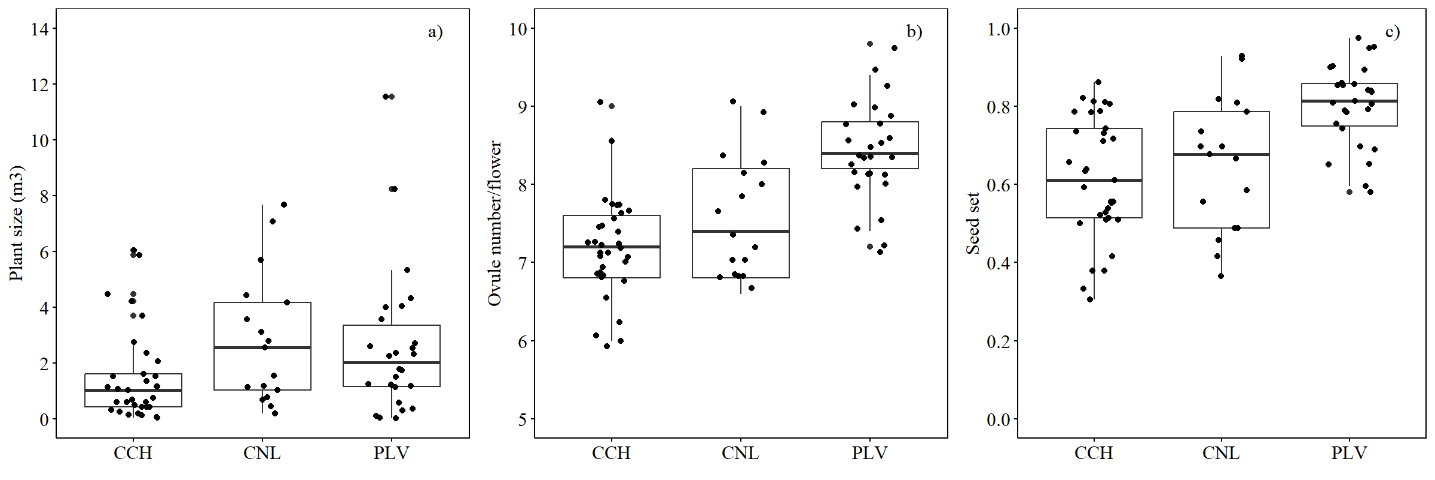
**

**FIGURE S1.** Boxplots of Plant size (Volume m^3^), Ovule number per flower, and seed set, observed in the three populations surveyed of *Vasconcellea chilensis*. CCH: Conchillas; CNL: Conchalí; PLV: Puntilla Las Vacas.

**TABLE S1**. Results of the Variance Component Analysis carried out on a) the number of ovules and b) the number of seeds observed in *Vasconcellea chilensis. “*Population” included variation among the three populations studied. Total mean ovule/flower = 7.7; N=455. Total mean seed/fruit = 5.7; N=512.

a)

| Variation source | df | SS | MS | Variance component | % | SD | CV [%] |
| --- | --- | --- | --- | --- | --- | --- | --- |
| Total | 16.1 |  |  | 2 | 100 | 1.4 | 18.2 |
| Population | 2 | 198.5 | 99.2 | 0.7 | 34.1 | 0.8 | 10.6 |
| Pop./plant | 92 | 231.4 | 2.5 | 0.3 | 16.2 | 0.6 | 7.3 |
| Error | 360 | 353 | 1 | 1 | 49.7 | 1 | 12.8 |

b)

| Variation source | df | SS | MS | Variance component | % | SD | CV [%] |
| --- | --- | --- | --- | --- | --- | --- | --- |
| Total | 15.4 |  |  | 4,6 | 100 | 2.1 | 37.7 |
| Population | 2 | 545.7 | 272.9 | 1.6 | 34.3 | 1.3 | 22 |
| Pop./plant | 83 | 792 | 9.5 | 1.3 | 28.3 | 1.1 | 20 |
| Error | 426 | 739.7 | 1.7 | 1.7 | 37.4 | 1,3 | 23 |

**TABLE S2.** Correlation matrices for independent variables used in the linear models fitted for seed set (dependent variable) of *Vasconcellea chilensis* in the surveyed populations. CCH: Conchillas; CNL: Conchalí; PLV: Puntilla Las Vacas. Independent variables; NMD: Nearest Neighbor Distance; SMD: Sum of Male Distances; VOL: Plant Volume

| Site: CCH | NMD | SMD | VOL |
| --- | --- | --- | --- |
| NMD | 1 | 0.74, P<0.01 | 0.16, P=0.4 |
| SMD |  | 1 | 0.30, P=0.08 |
| VOL |  |  | 1 |

| Site: CNL | NMD | SMD | VOL |
| --- | --- | --- | --- |
| NMD | 1 | -0.49, P=0.04 | 0.32, P=0.2 |
| SMD |  | 1 | -0.5, P=0.03 |
| VOL |  |  | 1 |

| Site: PLV | NMD | SMD | VOL |
| --- | --- | --- | --- |
| NMD | 1 | 0.03, P<0.9 | 0.004, P=0.9 |
| SMD |  | 1 | -0.26, P=0.19 |
| VOL |  |  | 1 |

**TABLE S3.** Values of Variance Inflation Factor (VIF) observed for each independent variable used in the initial linear models, fitted on a seed set (dependent variable) of *Vasconcellea chilensis.* When a VIF value was higher than 1/1-R^2^, the variable (with the highest VIF) was removed from the model. Independent variables: NMD: Nearest Neighbor Distance; SMD: sum of Male Distances; VOL: Plant Volume. CCH: Conchillas; CNL: Conchalí; PLV: Puntilla Las Vacas.

| Population: CCH | VIF | R^2^ | 1/1 – R^2^ |
| --- | --- | --- | --- |
| NMD | 2.2 | 0.23 | 1.3 |
| SMD | 2.4 |  |  |
| VOL | 1.1 |  |  |

| Population: CNL | VIF | R^2^ | 1/1 – R^2^ |
| --- | --- | --- | --- |
| NMD | 1.3 | 0.14 | 1.2 |
| SMD | 1.6 |  |  |
| VOL | 1.4 |  |  |

| Population: PLV | VIF | R^2^ | 1/1 – R^2^ |
| --- | --- | --- | --- |
| NMD | 1 | 0.14 | 1.8 |
| SMD | 1.1 |  |  |
| VOL | 1.1 |  |  |

**TABLE S4.** Regression coefficients of the final Linear Models fitted to examine the seed set of *Vasconcellea chilensis,* in the three surveyed populations (in bold). Independent variables: VOL: Plant volume; NMD: Nearest male neighbor distance; SMD: Sum of Male distances.

|  | Estímate | Std. Error | t value | P |
| --- | --- | --- | --- | --- |
| **Conchillas** |  |  |  |  |
| Intercept | 0.6908 | 0.0391 | 17.66 | <0.001 |
| VOL | 0.0076 | 0.0146 | 0.52 | 0.6068 |
| NMD | -0.0036 | 0.0010 | -3.451 | 0.0017 |
| **Conchalí** |  |  |  |  |
| Intercept | 0.6985 | 0.0878 | 7.9600 | <0.001 |
| VOL | -0.0061 | 0.0207 | -0.2960 | 0.7720 |
| NMD | -0.0039 | 0.0097 | -0.4030 | 0.6930 |
| **P. Las Vacas** |  |  |  |  |
| Intercept | 0.769 | 0.128 | 6.004 | <0.001 |
| VOL | 0.005 | 0.004 | 1.120 | 0.274 |
| NMD | -0.013 | 0.003 | -4.089 | <0.001 |
| SMD | 0.001 | 0.001 | 0.663 | 0.514 |
